# Principles of Human Physiology, with Their Chief Applications to Pathology, Hygiene, and Forensic Medicine

**Published:** 1843-07-01

**Authors:** 


**Principles of Human Physiology, with their chief Applications to Pathology, Hygiene, and Forensic Medicine. Especially designed for the use of Students. By *William B. Carpenter*, M.D. Lecturer on Physiology in the Bristol Medical School, &c. London, Churchill, 1842. pp. 680.**

The elementary treatises on Physiology published in this country of late years, have scarcely brought their information up to the level of the science which existed even at the time of their first appearance: and the rapid progress of physiological knowledge has now left them far in the rear. The works of Bostock, Elliotson, Mayo, and others, though containing much valuable matter, scarcely touch upon certain facts recently established, and views recently divulged, which at present give, to the whole science of life, its principal impulse and direction. Dr. Carpenter's book, therefore, has not made its appearance uncalled for, while its peculiar character would have rendered it valuable, even though the sources of elementary information for the English student of physiology had been more abundant than they are. It might be thought that the excellent English versions of the first German physiologists which have either been recently completed, or are now in progress—as those of Müller and Wagner—would have supplied the deficiency referred to. But it will be found on examination, that these works, though of a higher scientific cast than the treatise before us, are not as well calculated for the use of the student—the profound and original views, and elaborate detail of the minutest facts with which they abound, rendering them much more profitable to the mature physiologist, than to one only just entering on the same grand but bewildering field of research. Dr. Carpenter's work is, in particular, much better adapted to the medical student than any other of the kind that we have met with, in as much as its plan has a direct reference to medicine, and the bearings of physiology on the various branches of that science and art are set forth in a philosophical and lucid manner.

Dr. Carpenter introduces his subject by a general view of the connexion of physiology with the other branches of medicine. This introduction cannot be too strongly recommended to the student. It contains a very able and comprehensive yet simple exposition of the relations of the science of life to medicine in general—of the dependencies of the different branches of medicine on each other—and of the relative value of the practical conclusions derived from scientific principles, and from direct experience.

Chapter I. treats of the place occupied by man in the scale of being, and involves the consideration of the distinction between animals and plants—the general subdivisions of the animal kingdom—the characters of its four primary groups—and the characteristics of man. All these subjects are treated with accuracy and judgment. The following is an ingenious addition to the various attempts at forming a precise line of demarcation between animals and vegetables.

"A distinction might probably be erected between the animal and vegetable kingdoms, upon the mode in which the first development of the germ takes place. The seed of the plant at the time of fertilisation, principally consists of a store of nourishment prepared by the parent for the supply of the germ, which is introduced into the midst of it. The same may be said of the egg of the animal. In both instances, the first development of the germ is into a membranous expansion, which absorbs the alimentary materials with which it is in contact, and prepares them by assimilation for the nourishment of the embryonic structure, the most important parts (in the higher classes of animals, and in phanerogamic plants, the only permanent parts) of which are in its centre. Now, in plants, this membranous expansion (the single or double cotyledon) absorbs by its outer surface, which is applied to the albumen of the seed, and takes it more or less completely into its own substance. In animals, this expansion is developed in such a manner, that it surrounds the albumen, enclosing it in a sac, the *inner* surface only of which is concerned in absorption. This sac is, then, the temporary *stomach* of the embryonic structure; it becomes the permanent stomach of the radiata; but in the higher classes only a portion of it is retained in the fabric of the adult,—the remainder being cast off, like the cotyledon of plants, as soon as it has performed its function. Thus, then, the first *nisus* of animal development is towards the formation of a stomach, for the internal reception and digestion of food, whilst the first processes of vegetable evolution tend to the production of a frond-like membrane, which, like the permanent frond of the lower classes of plants, absorbs nourishment by its expanded surface only." 12.

This distinction obtains uniformly till we descend to the lowest degrees of the animal scale; but here we meet with an exception in the early condition of the branching asteroida, which, before the development of the polype-cells, appear to be nourished by absorption throughout their surface, but which, though not yet perfectly developed, are, nevertheless, unquestionably animals. It is not improbable that the attempts to discriminate between the essential nature of animals and plants may lead to results widely different from those with a view to which they have been instituted.

It is nearly certain that all organised bodies, whether animal or vegetable, originate from a similar germinal spot, and are at first developed by a like process—the successive evolution of cells, The more we become acquainted with the organisation of animals and plants, the more points of similarity do we discover among them; while, with reference to the lowest types of presumed animal existence—the porifera—the further we investigate them, the less ground do we find for certainty as to which kingdom they belong to.

These considerations would rather incline us to infer, that animal and vegetable life are not separated by any exact boundary, and that certain beings, low in the scale of organisation, not only appear to be, but actually are, connecting links between them.

Chapter II. embraces a general view of the functions of organised bodies based upon the important physiological truth, that the state of life, in whatever form, is one of perpetual change. The grand distinction between an inorganic and a living body is this: an inorganic body, in order to preserve its existence in the same form, must be exempted from the impression of surrounding bodies;—in other words, it must remain unchanged; a living body, on the contrary, in order to preserve its existence in the same form, must be kept in a state of perpetual relation to surrounding bodies, and must be constantly impressed by them so as to undergo continual changes. The one remains both apparently and substantially the same body; the other continues apparently the same, but is not substantially so at any two periods of its existence, which existence is in effect a series of continuous repetitions of itself, each repetition deviating, in an imperceptible degree, through the various stages of growth, development, maturity, and decay, till the susceptibility of reaction—the condition of the state of vital relation—is abolished, and the phenomena of life and the existence of the individual organism cease along with it. Dividing the functions of the human body into those of vegetative and those of animal life, our author gives a perspicuous general view of each, into which it is not necessary that we should here enter.

Chapter III. is on the functions of the nervous system, and evinces, in a remarkable degree, the extensive information and sound understanding of the author.

Nothing can be more just, guarded, or appropriate than the following remarks on the sensorial functions—a subject on which physiological writers in general are extremely apt to commit themselves.

"It is well to explain that, though the physiologist speaks of the intellectual powers, moral feelings, &c. as *functions* of the Nervous System, they are not so in the sense in which the term is employed in regard to other operations of the bodily frame. In general, by the *function* of an organ, we understand some change which may be made evident to the senses, as well in our own system as in the body of another. Sensation, Thought, Emotion, and Volition, however, are changes imperceptible to our senses, by any means of observation we at present possess. We are cognisant of them in ourselves, without the intervention of those processes by which we observe material changes external to our minds; but we judge of them in others, only by inferences founded on the actions to which they give rise, when compared with our own. When we speak of sensation, thought, emotion, or volition, therefore, as functions of the Nervous System, we mean only that this system furnishes the conditions under which they take place in the living body; and we leave the question entirely open, whether the ψυχη has or has not an existence independent of that of the material organism, by which it operates in Man as he is at present constituted." 80.

In this chapter Dr. Carpenter gives an excellent view of the elementary structure of the nervous system—of the elementary functions of nervous structure—of the mode of determining the functions of nerves—of the nature of the changes which take place in the nerves when they receive and transmit impressions—of the comparative anatomy and physiology of the nervous system—and of the structure and functions of the same system in the vertebrata, and especially in man. The physiological anatomy of the cerebro-spinal axis is here detailed with greater fulness and accuracy than in any English work with which we are acquainted. The chapter concludes with a general recapitulation and pathological applications which we wish we could transcribe entire. The following, which is all for which we can find space, will convey an idea of Dr. Carpenter's comprehensive and lucid manner of treating such subjects.

"A general Summary of the views here propounded, in regard to the Functions of the Cerebro-Spinal division of the Nervous system, may probably be useful in assisting the Student to gain clear ideas regarding them.—The fibres of the nervous trunks may be divided, according to the direction of their influence, into two classes,—the *afferent* or *centripetal*,—and the *efferent* or *centrifugal*. The afferent may be said to commence at the periphery, especially on the skin, mucous surfaces, &c., and to terminate in the grey matter of the nervous centres; whilst the efferent originate in that grey matter, and terminate in the muscles.* Every fibre runs a distinct course from its origin to its termination; and it is not improbable that the endowments of the different fibres composing each trunk are very distinct. From the great vascularity of the grey matter, and the occurrence of a structure of corresponding character around the origins of the afferent nerves, it is evident that its functions must be different from those of the fibrous structure; and, whilst there is no evidence that the latter serves any different purpose than that of a mere conductor, there seems good reason to believe that all the active operations, of which the nervous system is the instrument, originate in the former. A mass of grey matter connected with nervous trunks, forms a *ganglion;* and in the Invertebrata, the ganglia are frequently numerous, and are scattered through the system, without much connection with each other,—each having a distinct function. In Vertebrate animals, on the other hand, they are united into one mass,—partly, it would seem, for the sake of the protection afforded them by the bony skeleton,—and partly in order that more complete consentaneousness of action may be attained. Still, several distinct divisions may be traced in the centres of the cerebro-spinal system,—partly by the determination of their respective functions, as indicated by observation and experiment,—and partly by the study of the distribution of the nerves proceeding from them. In this manner we arrive at the knowledge of several distinct ganglionic centres, of which the following may be considered as a general account.

I. The True Spinal Cord, consisting of a nucleus of grey matter, receiving afferent fibres, and giving origin to efferent; by these it is connected with all parts of the body, but especially with the surface and muscles of the extremities. The actions of this centre may be performed without consciousness on the part of the individual; and they consist in the reflexion of a motor impulse along an efferent nerve, on the reception of a stimulus conveyed by an afferent or excitor nerve. These reflex movements can be best excited, when the muscles are removed from the control of the will, which otherwise would generally antagonise them. Some of them are connected with the maintenance of the organic functions; and others with the protection or withdrawal of the body from injury. Muscular movements may also be excited by a stimulus directly applied to the Spinal Cord itself.

II. The Medulla Oblongata, or cranial prolongation of the Spinal Cord. The actions of this do not essentially differ from those of the true Spinal Cord; but they are connected with different organs. This part consists chiefly of the centres of the nerves of Respiration and Deglutition,—two functions of which the continual maintenance is essential to the life of the being; and it would seem as if these were placed within the cranium, to be more secured from accidental injury. The movements concerned in Respiration and Deglutition are, like those excited through the true Spinal Cord, of a strictly reflex character, being in all instances due to an impression originating in the periphery of the system, which,

---

* "In neither case, however, can the word *terminate* be used with strict correctness; since there is good reason to believe that the apparent termination is not real, but that the ultimate fibres spread from each other in loops, so as to form a plexus, in which there is no loss or cessation of any one of them."

being conveyed to the centre, excites there a motor impulse; and they, also, are independent of Sensation.

III. The Ganglia of the nerves of Special Sensation, which form, as it were, the continuation of the Medulla Oblongata. These, also, appear to minister to actions, which do not differ widely from the Reflex in character,—being almost necessarily excited by certain stimuli, and being only in a degree controllable by the will. But their actions differ in this,—that they are attended with consciousness, and also, it would appear, with certain peculiar feelings. Reasons have been given for the belief, that these ganglia are the centres of those actions, which are commonly termed *instinctive* in the lower animals, and *consensual* and *emotional* in ourselves; these all correspond, in being performed without any idea of a purpose, and without any direction of the will,—being frequently in opposition to it.

IV. The Cerebral Hemispheres or ganglia, which are evidently the instruments or organs of the intellectual faculties. These are connected, by fibres of communication, with almost all parts of the body; and from their proportional size in Man, it seems probable, that many of the nervous trunks are principally composed of such fibres. It is probably by them alone, that ideas or notions of surrounding objects are acquired, and that these ideas are made the ground-work of mental operations. They would seem, also, to be the exclusive seat of Memory. The results of these operations are manifested on the bodily frame, through the Will, which is capable of acting in greater or less degree, on all the muscles forming part of the system of Animal life.

V. The Cerebellum, which appears to be concerned in the regulation and harmonisation of Muscular movement, whether Instinctive or Voluntary.

*Tabular View of the Nervous Centres.*

| | | |
|---|---|---|
| | Cerebral Ganglia, the centres of the operations of intelligence and will. | |
| Nerves of special sensation.— Motor fibres mingled with general motor system. | Ganglia of Special Sense, the centres of consensual, instinctive, and emotional actions. | Nerves of special sensation. —Motor fibres mingled with general motor system. |
| | Cerebellic Ganglia, for harmonisation of general muscular actions. | |
| Afferent and Motor Nerves of Respiration, Deglutition, &c. | Repiratory and stomato-gastric Ganglia. | Afferent and Motor Nerves of Respiration, Deglutition, &c. |
| Trunks of Spinal nerves, composed of fibres from true Spinal Cord, and from Cerebrum, Cerebellum, and Medulla Oblongata; each group containing afferent and efferent fibres. | Fibrous structure, continuous with Brain. / True Spinal Cord, consisting of chain of ganglia, for Reflex actions of the trunk and extremities. / Fibrous structure, continuous with Brain. | Trunks of Spinal nerves, composed of fibres from true Spinal Cord, and from Cerebrum, Cerebellum, and Medulla Oblongata; each group containing afferent and efferent fibres. |

CHAPTER IV. is on sensation and the organs of the senses. The section on "Sensation in general" is written in a highly philosophical manner. It concludes with a reference to the curious observations of Dr. Henry Holland, relative to the effects of the continued direction of attention on particular parts of the body, as modifying not only their sensations, but, ultimately, also their organic actions. We may notice, in passing, another set of phenomena, of a kindred nature, too little attended to by physiologists; namely, the effects of intense sensorial action in exalting the functions of the nerves of special sensation, or at least in exalting the senses themselves—for this may be conceived to arise either from an increase of sentient power in the nerve, or of perceptive power in the brain. For example, it often happens that a person intensely interested in an event, will clearly discern an object which is beyond their ordinary limit of vision; and, in like manner, fear or anxious suspense will render the ear cognisant of sounds so faint, that they would not, under ordinary circumstances, have been perceived.

The sections on Vision and Audition show Dr. Carpenter to be well versed in the branches of natural philosophy, on the principles of which the exercise of those senses depends.

On the cause of erect vision from an inverted image on the retina, he expresses himself as follows :—

"Many solutions of it have been attempted; but they are for the most part rather specious than really satisfactory. That which has been of late years the most in vogue, is founded upon what was styled the Law of Visible Direction, which has been supported by Sir D. Brewster, and other eminent Philosophers. This law affirms, that every object is seen in the direction of the perpendicular to that point of the retina, on which its image is formed; or, in other words, that, as all the perpendiculars to the several points of the inner surface of a sphere meet in the centre, the line of direction of any object is identical with the prolonged radius of the sphere, drawn from the point at which its image is made upon the retina. Upon close examination, however, it is found that this law cannot be optically correct; since the lines of direction cross each other at a point much anterior to the centre of the globe; as may be determined by drawing a diagram upon a large scale, and laying down the course of the rays received by the eye, according to the curvatures and refractive powers of its different parts. In this manner it has been determined by Volkmann, that the lines of direction cross each other in a point a little behind the crystalline lens; and that they will thus fall at such different angles on different points of the retina, that no general law can be laid down respecting them. It may be questioned, moreover, whether any such law would afford any assistance in explaining the phenomenon; since, after all, it is requisite to assume an intuitive application of it, in supposing the mind to derive its ideas of the relative situations of objects from the supposed line of direction. A much simpler and more direct explanation may be given. We must remember that which we have had occasion to notice in regard to all the other senses,—the broad line of distinction between the sensation and the perception or elementary notion; and this is still more clearly shown by the complete absence of any relation, but such as experience developes, between the perceptions derived through the sight, and those acquired from the touch. Hence there is no more difficulty in understanding, that an inverted picture upon the retina should convey to us a notion of the external world, which harmonises with that acquired through the sense of touch, than there is in comprehending the formation of any of those intuitive perceptions of animals, which are so much more removed from the teachings of our own experience.

It is justly remarked by Müller that, 'if we do see objects inverted [or rather, if the picture on the retina is inverted] the only proof we can possibly have of it, is that afforded by the study of the laws of Optics; and, if everything is seen reversed, the relative position of the objects remains unchanged. Hence it is, also, that no discordance arises between the sensations of inverted vision and those of touch, which perceives everything in its erect position; for the images of all objects, even of our own limbs, on the retina, are equally inverted, and therefore maintain the same relative position. Even the image of our hand, when used in touch, is inverted.' From what has been stated it would appear quite conceivable, that a person just endowed with sight, should not at first know by his visual powers, whether a pyramid placed before his eyes is the same body, and in the same position, as one with which he has become acquainted by the touch; and, if this be admitted, the inference necessarily follows, that the notion of *erectness*, which we form by the combined use of our eyes and our hands, is really the product of experience in ourselves, whilst it is probably innate or intuitional in the lower animals." 266.

It seems to us that one of the best solutions of this difficulty hitherto offered, is that framed by Sir Charles Bell on Brown's doctrine of the Muscular Sense. According to this theory, we determine whether an entire object be above or below us, to the right hand or to the left, by our consciousness of the action of that muscle which directs the eye towards the object. In like manner we distinguish the top of an object from the bottom by a consciousness of the action of the levator muscle when we regard the former, and of the depressor when we regard the latter.

It may be objected, that this view will not apply in the case of objects so minute as to require no muscular action to enable the eye to apprehend them, but which notwithstanding, are seen in their true position as well as larger objects. But this objection is invalid; because, although no muscular action be required to enable the eye to take in such objects, yet the smallest elevation of the eye banishes the object, and the point which disappears last is thus known to be the top; while the smallest depression of the eye also banishes the object, and the point which disappears last is thus known to be the bottom; and so on with regard to the movements of the eye from right to left, and left to right, or in any other direction.

It is probable that several causes concur in the production of the result in question; but we have little doubt that Sir Charles Bell's view is at least applicable to a considerable extent. We should be much disposed, also, to refer our notions of *magnitude*, in many instances, to the same cause.

Doubtless, we acquire information on this head from the size of the image formed on the retina; the degree of illumination of the object, and the habit of comparing objects when seen together. Still, it appears to us that these circumstances alone are inadequate to account for our perceptions of magnitude, and that we are greatly assisted in this respect by a consciousness of the degree and duration of muscular action necessary to carry the eye over the surface of an object, or with reference to extremely minute objects, by the consciousness that no such action is required, and that the smallest movement of the eye diverts it altogether from the object. With regard to objects of considerable, but unequal dimensions, placed at the same distance from the observer, we think it probable that the estimate of magnitude is formed almost entirely by the exercise of the muscular sense.

CHAPTER V. treats of muscular contractility. This, like every other subject comprehended in the work, has been ably handled by Dr. Carpenter, but, as it is one on which no great accession of knowledge has been obtained of late years, we pass it by.

CHAPTER VI. is on the voice and speech, and contains an interesting view of the formation of the voice in speech and song, and of the principle and construction of musical instruments, derived chiefly from the investigations of Willis, Müller, and others. The section on Articulate Sounds is a good and practical one, and concludes with the following remarks on stammering:—

"Great as is the number of muscles employed in the production of definite vocal sounds, the number is much greater for those of articulate language; and the varieties of combination, which we are continually forming unconsciously to ourselves, would not be suspected without a minute analysis of the separate actions. Thus, in uttering the explosive sounds, we check the passage of air through the posterior nares, in the very act of articulating the letter; and yet this important movement commonly passes unobserved. We must regard the power of forming the several articulate sounds which have been adverted to, and their simple combinations, as so far resulting from intuition, that it can in general be more readily acquired by early practice than other actions of the same complexity; so that we may consider these movements as having somewhat of the same consensual character as that which has been attributed to the purely vocalising actions. But there is in many individuals a deficiency of the power of rightly combining them, from which stammering and other imperfections result. Many theories regarding the nature of stammering have been proposed; and there can be little doubt that the impediment may be attributed to a great variety of exciting causes. A disordered action of the nervous centres must, however, be regarded as the proximate cause; though this may be (to use the language of Dr. M. Hall) either of *centric* or of *excentric* origin,—that is, may result from a morbid condition of the ganglionic centre, or from an undue excitement conveyed through its afferent nerves. When of centric origin (and this is probably the most general case) the phenomena of stammering and chorea have a close analogy to each other; in fact, stammering is frequently one of the modes, in which the disordered condition of the nervous system in chorea manifests itself. It is in the pronunciation of the consonants of the explosive class, that the stammerer experiences the greatest difficulty. The total interruption to the breath which they occasion frequently becomes quite spasmodic; and the whole frame is thrown into the most distressing semi-convulsive movement, until relieved by expiration.* In the pronunciation of the continuous consonants of the first class, the stammerer usually prolongs them, by a spasmodic continuance of the same action; and there is, in consequence, an impeded, but not a suspended respiration. The same is the case with the *l* and *r* in the second class. In pronouncing the *m* and *n*, on the other hand, as well as the aspirates

* "By Dr. Arnott this interruption is represented as taking place in the larynx; that this is not the case, a little attention to the ordinary phenomena of voice will satisfactorily prove."

and vowels, it is sometimes observed that the stammerer prolongs the sound, by a full and exhausting expiration. In all these cases, then, it seems as if the muscular sense, resulting from each particular combination of actions, became the stimulus to the involuntary prolongation of that action. In some instances it is possible that the defect may result from malformation of the parts about the fauces producing an abnormal stimulus of this kind in some particular positions of the organ; and such cases *may be* really benefited by an operation for the removal of these parts. But the effect of the operation is evidently for the most part upon the nervous system; and it coincides with what may be frequently observed,—that the stammering is increased under any unusual excitement, especially of the emotional kind.

"The method proposed by Dr. Arnott for the prevention of stammering, consists in the connexion of all the words by a vocal intonation, in such a manner that there shall never be an entire stoppage of the breath. It is justly remarked by Müller, however, that this plan may afford some benefit, but cannot do every thing, since the main impediment occurs in the middle of words themselves. One important remedial means, on which too much stress cannot be laid, is to study carefully the mechanism of the articulation of the difficult letters, and to practise their pronunciation repeatedly, slowly, and analytically. The patient would at first do well to practise sentences, from which the explosive consonants are omitted, and his chief difficulty, arising from the spasmodic suspension of the expiratory movement, would be thus avoided. Having mastered these, he may pass on to others in which the difficult letters are sparingly introduced, and finally accustom himself to the use of ordinary language. One of the chief points to be aimed at, is to make the patient feel that he has command over his muscles of articulation; and this is best done by gradually leading him from that which he finds he *can* do, to that which he fears he cannot. The fact that stammering people are able to *sing* their words better than to *speak* them, has been usually explained on the supposition that, in singing, the glottis is kept open, so that there is less liability to spasmodic action; if, however, as here maintained, the spasmodic action is not in the larynx, but in the velum palati and the muscles of articulation, the difference must be due to the direction of the attention rather to the muscles of the larynx than to those of the mouth. Every one must have noticed how much the impediment of stammerers is increased, when they are particularly anxious to speak fluently." 338.

Chapter VII. treats of the influence of the nervous system on the organic functions. It is brief, being evidently intended rather to connect other portions of the work than to exhaust the subjects which are commented upon.

Chapter VIII. is on Digestion and Absorption. Little that is new can be expected on these heads; our author has, however, given a correct view of the present state of knowledge in regard to them, with judicious dietetic and hygienic applications.

Chapter IX. is devoted to the all-important subject of the Circulation, and is divided into sections relating to the action of the heart—the causes influencing the circulation in the arteries and capillaries—the venous cirlation—and the peculiarities of the circulation in particular parts. The most important, as well as the most obscure, department of the subject, in the present state of physiological research, is that relating to the vital endowments of the capillaries and their influence on the passage of the blood through them.

But a few years since, and the capillaries were generally regarded as active agents in the propulsion of the blood, while, by a singular confusion of ideas, they were at the same time regarded as the immediate seat of the ultimate processes of life. But, recent researches into the physical phenomena of life, and the rapid progress of microscopical anatomy, have dispossessed us of both these errors, and induced the belief that the capillary vessels, for the most part, simply conduct the materials for processes which are extra-vascular. The adoption of this belief, however, has led, in many quarters, to a depreciation of the importance of capillary action in the regulation of vital processes. We regard it as sufficiently established that the capillary vessels have no general influence in propelling the blood and that they merely convey it as the material of vital actions over which they can have no immediate control. But have they no indirect influence on these actions? Is it a question of no importance whether they do, or do not, by a vital power of varying their diameter, affect the *distribution* of the blood, and its *velocity* in particular vessels—for, according to the common law of hydraulics, its velocity must, *cæteris paribus*, be greater in a narrower than a wider tube? On this question, be it observed, are contingent, not only with what surfaces the immediate materials of vital action shall principally be brought in contact, but, also, the quantity of such materials which shall be subjected to a given vital agency in a given time—which amounts virtually to the *proportion* of certain ingredients in a given vital process.

Dr. Carpenter's views of the subject do not precisely agree with those here expressed; but they differ only with respect to points on which, in the present state of knowledge, our notions must necessarily be too vague to be worth disputing about. The following passage we regard as conveying a correct practical view of the agency of the capillaries.

"Many of the facts which indicate the influence of the capillaries on the amount and rapidity of the circulation through them, have been already adverted to. It is a general fact, unquestioned by any physiologists that, when there is any local excitement to the processes of nutrition, secretion, &c., a determination of blood towards the part speedily takes place, and the motion of blood through it is increased in rapidity; and although it might be urged that this increased determination may not be the effect, but the cause, of the increased local action, such an opinion could not be sustained without many inconsistencies with known facts. For it is known that such local determination may take place, not only as a part of the regular phenomena of growth and development (as in the case of the entire genital system at the time of puberty and of periodical heat, the uterus after conception, and the mammæ after parturition), but also as a consequence of a strictly-local cause. Thus the student is well aware that, after several hours' close application, there is commonly an increased determination of blood to the brain, causing a sense of oppression, a feeling of heat, and frequently a diminished action in other parts; and, again, when the capillary circulation is being examined under the microscope, it is seen to be quickened by moderate stimuli, and equally retarded by depressing agents. All these facts harmonise completely with the phenomena which are yet more striking in the lower classes of organised beings, and are evidently the results of the same laws.

"If the phenomena which have been here brought together be considered as establishing the existence, in all classes of beings possessing a circulating apparatus, of a capillary power, which affords a necessary condition for the movement of the nutritious fluid, through those parts in which it cames into more immediate relation with the solids,—the question still remains open, as to its nature. That the capillaries possess a contractile power, in a far higher degree than do the large arteries, and more easily excited than that of the smaller, appears scarcely to admit of doubt; though to what it is due, may be reasonably questioned. It has been recently asserted by Schwann, that they possess the same kind of fibrous tissue in their walls as do the large vessels: and this cannot be regarded as improbable. It is not possible, however, that their contractility could have any influence in aiding the continuous motion of blood through them, unless it were exercised in a very different manner from that of which observation affords us evidence. For, when we are microscopically examining the capillary circulation of any part, it is at once seen that the vessels present no obvious movement, and that the stream, now rendered continuous by the elasticity of the arteries, passes through them as through unelastic tubes. The only method in which the contractility of the capillaries could produce a regular influence on the current of blood, would be an alternate contraction and dilatation, or a peristaltic movement; and of neither of these can the least traces be discerned. Hence we should altogether dismiss from our minds the idea of any *mechanical* assistance afforded by the action of the capillaries to the movement of the blood. That the contractile coat of the capillaries has for its office to regulate the calibre of the vessels, can scarcely be doubted; but any general permanent contraction would only occasion an obstacle to the circulation,—as is shown by the effects of stimulating injections, which, if thrown into the vessels before their vitality has been lost, will not pass through the capillaries. It would appear, therefore, to be through their action on this coat, that local stimuli occasion a contraction of the capillaries; their effect, however, is different from what might have been anticipated; for, instead of the capillary circulation being retarded, it is accelerated, at least until an abnormal condition results from their continued operation. Here, again, is another evidence, that something different from mechanical power must be the agent that operates in all the foregoing cases." 416.

Chapter X. is on Respiration, and contains a good exposition of the facts of the subject, on which we have no particular observations to make.

Chapter XI. on the subject of Nutrition, takes a wide range, and embraces a number of the most important points in general physiology.

These are considered under the heads of, Organisable Principles—the Formation of Cells—the Elaboration of Chyle and Lymph—the Physical and Vital Properties of the Blood—Pathological Changes in the Blood—the Origin of the Solid Tissue—and the Formation of the Tissues.

Dr. Carpenter's observations on these subjects form a good critical review of the statements of physiologists on points concerning many of which our knowledge is recent and very imperfect. He has wisely abstained from controversy, and been content with the enunciation of what has either been ascertained or rendered probable. The extent of the field precludes our entering into any of the subjects adverted to, nor indeed, does our author's method of treating them call for any special remarks on our part. We are in a similar position with respect to,

Chapter XII. in which the function of secretion is considered under the heads of Secretion in General—Secretion of Bile—of Urine—of Milk—that of the Salivary Glands and Pancreas—of the Lachrymal Gland

—of the Testis—of the Cutaneous and Mucous Follicles—the Functions of the Spleen and Supra-renal Capsules—and of the Thymus and Thyroid bodies, which, from their general aspect, and our ignorance of their precise office in the animal economy, are usually associated with the glandular organs.

These subjects are handled by Dr. Carpenter in a manner which shows his complete knowledge of physiology in all its departments, and his philosophic disposition to adhere to facts and legitimate deductions—not hazarding hypotheses for the sake of an ephemeral reputation for ingenuity.

In CHAPTER XIII. we find a general survey of the nutritive processes, with some practical applications to medicine, and a review of the subject of animal heat. This chapter is very ably written, and affords evidences of extensive and well-digested information. As an example of our author's applications of physiology to medicine, we subjoin the following extract:

"There are many disorders commonly regarded as affections of the Circulation, which evidently consist in reality of a morbid alteration in the Nutritive processes; among these, there can be little doubt that we are to rank Inflammation. Much has been said and written, to very little purpose, respecting the essential nature of this process; it has been attributed by some to disordered action of the vessels, and by others to an injurious impression on the nerves,—the fact, that inflammation may occur in tissues which contain neither vessels or nerves, having been entirely overlooked. The only view of the character of Inflammation that seems likely to account for its phenomena, is that which regards it as essentially consisting in a disturbance of the due relation between the living tissue and the nutrient materials contained in the blood. As to the nature of this disturbance, we know no more than we do of the nature of the relation itself; and the expression must, for the present, have somewhat of a vague and indefinite character. Nevertheless, it is much better to have a dim vision of the true beacon, than to be led astray by the more attractive glare of false lights; and whilst the specious hypotheses, which profess to make the whole subject easy of comprehension, are found to be more fallacious the more they are examined into,—the one just adverted to becomes more satisfactory, the more it can be connected with precise data. That there must be a certain relation or adaptiveness, between the substance of the tissues, and the materials at whose expense they are formed, appears sufficiently evident. It has been pointed out that the albumen of the blood is converted, during its circulation, into fibrin; and that the fibrin is withdrawn and assimilated by the solid parts. In the tuberculous cachexia, it has been shown that the fibrin is deficient, and that the tissues are consequently nourished but imperfectly, whilst unorganisable albumen is deposited amongst them. On the other hand, in the inflammatory diathesis, there is probably an increased tendency in the blood to the generation of fibrin; and this, by disturbing the due relation between the nutritive fluid and the solid tissues, may become a cause of local disease,—the morbid action which results from this condition of the blood being determined to a particular part by some extraneous causes. In this morbid action, fibrinous matter is effused, either into the substance of the tissue affected, or upon its surface; there is a tendency to organisation, but in both cases its degree may vary,—a perfectly-formed tissue being produced, or a degeneration taking place into pus-globules, according to circumstances. Inflammation may result, however, from causes purely local, and primarily affecting the solid tissues; here, therefore, the disturbance of the normal relation is on the other side, yet the production of an increased amount of fibrin is still a character of the disease. Whether the blood moves faster or slower in an inflamed part,—whether the capillaries are contracted or dilated,—are questions, therefore, of little moment, in comparison with those which affect the actions of Nutrition and Secretion, to which the fluid, in its passage through the parts in question, ought to be subservient. The same may be remarked of those productions which have been termed *heterologous* transformations of tissue; but which are rather to be regarded as new growths, that have appropriated the nutriment designed for the support of the proper tissues, and have therefore become developed at the expense of these. It is quite as absurd to attempt to account for the growth of Schirrus, Carcinoma, &c., by any peculiar action of the vessels of the part, as it would be to attribute the secretion of fatty matter by the cells of one tissue, or of phosphate of lime by those of another, to the peculiar distribution of their vessels. The progress of research obviously leads to the conclusion, that in every part of the living body there is an inherent and independent vitality, which enables it to grow and maintain its normal structure and constitution, so long as it is supplied with the requisite materials; and that changes in the character of the tissue can be referred to nothing else than to alterations in its properties, resulting from external agencies, or to alterations in the materials supplied for its renewal. Of these two morbific causes, the latter is undoubtedly the most frequent; and the tendency which is now gaining ground to seek in the Blood for indications of pathological changes, when there is an obvious general disturbance of the system, will probably lead to a greatly-increased knowledge of the real nature of diseased states; in spite of the opposition which any return to the Humoral Pathology is sure to excite, in the minds of those who regard it as an exploded and pernicious system." 601.

On the subject of animal heat, Dr. Carpenter does not assent to the prevailing chemical doctrine of the day, which assimilates calorification to combustion. He nevertheless takes a chemical view of the process which, in our opinion, is precisely that justified by the facts which are known. "At present," he says, "it may be stated as a general fact, that the production of animal heat is due to the various changes in chemical composition that are continually taking place within the system; of which changes, the absorption of oxygen, and the disengagement of carbonic acid, are the two chief external manifestations:—and that the degree of caloric liberated bears a close relation to the activity of these changes, either in regard to the body at large, or to any portion of it." p. 611.

The concluding Chapter, on Reproduction and the Development of the Embryo, contains nothing original, but gives a succinct view of the actual amount of knowledge on these subjects.

We must now take our leave of Dr. Carpenter, and, in so doing we have much satisfaction in declaring our opinion that his work is the best systematic treatise on Physiology in our own language, and the best adapted for the student existing in any language.

